# Navigating the Semantic Labyrinth of “Sex” in the Study of Reproductive Trait Evolution

**DOI:** 10.1093/icb/icag093

**Published:** 2026-06-22

**Authors:** Caitlin E McDonough-Goldstein, Soleil E Young, Maurine Neiman, Sadye Paez, Nicole Valenzuela, Cibele G Sotero-Caio, Daniel L Jeffries, Octavio Manuel Palacios-Gimenez, Jessica K Abbott, Chiara Benvenuto, Ann Kathrin Huylmans, Sara Calhim, J Antonio Baeza, Andrew J Mongue, Erica L Larson, Lucija Andjel, Rainer Melzer, Elizabeth Dietz, Aurora Ruiz-Herrera

**Affiliations:** Department of Evolutionary Biology, University of Vienna, Vienna, 1030, Austria; Department of Integrative Biology, University of Wisconsin - Madison, Madison, WI 53706, USA; Plant and Microbial Sciences, University of California-Berkeley, Berkeley, CA 94720, USA; Department of Biology, University of Iowa, Iowa City, IA 52242, USA; Department of Gender, Women’s, and Sexuality Studies, University of Iowa, Iowa City, Iowa 52242, USA; Neurogenetics of Language Laboratory, The Rockefeller University, New York City, NY 10065, USA; Department of Ecology, Evolution, and Organismal Biology, Iowa State University, Ames, Iowa 50011, USA; Tree of Life Programme, Wellcome Sanger Institute, Hinxton, CB10 1RQ, UK; Institute of Ecology and Evolution, University of Bern, Bern, 3012, Switzerland; Population Ecology Group, Institute of Biodiversity, Ecology and Evolution, Friedrich Schiller Universität, Jena, 07743, Germany; Division of Biodiversity and Evolution, Biology Department, Lund University, Lund, 223 62, Sweden; School of Science, Engineering and Environment, University of Salford, Manchester, M5 4WT, UK; Institute for Organismic and Molecular Evolution (iomE), Johannes Gutenberg-Universität Mainz, Mainz, 55128, Germany; Department of Biological and Environmental Science, University of Jyväskylä, Jyväskylä, FI-40014, Finland; Department of Biological Sciences, Clemson University, Clemson, SC 29634, USA; Departamento de Biologia Marina, Universidad Catolica del Norte, Coquimbo, 1780000, Chile; Department of Entomology and Nematology, Institute of Food and Agricultural Sciences, University of Florida, Gainesville, Fl 32611, USA; Department of Biological Sciences, University of Denver, Denver, CO 80208, USA; Laboratory of Non-Mendelian Evolution, Institute of Animal Physiology and Genetics, The Czech Academy of Sciences, Liběchov, 277 21, Czech Republic; Department of Ecology, Faculty of Science, Charles University, Prague, 128 44, Czech Republic; School of Biology and Environmental Science, University College Dublin, Dublin, D04 V1W8, Ireland; Earth Institute, University College Dublin, Dublin, D04 V1W8, Ireland; Center for Biology and Society, Arizona State University, Tempe, AZ 85287,USA; Department de Biologia Cellular, Fisiologia i Immunologia, Universitat Autònoma de Barcelona, Cerdanyola del Vallès, 08193, Spain; Genome Integrity and Instability Group, Institut de Biotecnologia i Biomedicina, Universitat Autònoma de Barcelona, Cerdanyola del Vallès, 08193, Spain

## Abstract

“Sex” is a compound of many different concepts that contribute to a multiplicity of meanings. As a result, “sex” appears in a wide range of scientific research for different purposes, from describing a process of recombination and cell division to systems of categorising individuals. Identifying the meaning of “sex” in any particular context requires understanding the construction of contributing component concepts and their relationship with one another. We offer the metaphor of a labyrinth to describe the process of interpreting the meaning “sex” and uncovering its context-dependent purpose. In this way, the meanings of “sex” related to “sexual reproduction”, “sex types”, “sex determination”, and/or “sexual systems” can be elucidated by identifying how these concepts relate to suppositions about what properties of “recombination, “meiosis”, “syngamy”, or “gametes” are present and to which aspects of a trait, individual, or lineage they apply. By denoting “sex” and related terminology within quotation marks, we emphasise the contextualism of language in the theory and practise of research and resultant knowledge about the natural world. The structure of a labyrinth reflects the incredible capacity of “sex” to describe the diversity and disparity of eukaryotic reproductive strategies. The analytical framework of the labyrinth highlights that different conceptual versions can arise in the consideration of reproductive trait evolution based on the relationships among concepts. Through a critical evaluation of the concepts that contribute to the labyrinth of “sex” across biology, we gain a more nuanced appreciation for the operation of “sex” within scientific knowledge.

*“What if, when both animals and plants are sexed, “sex” becomes a wholly other thing?”*

- *Stella*

*Sandford (2022)*, *Vegetal Sex*

*“If you were a single-celled alga sitting in a pond, you wouldn’t see the world as splitting into males and females”*

- *Laurence Hurst quoted in*

*Whitfield (2004)*

## Introduction

“Sex” in biological research means many different things. However, “sex” is often treated as a self-evident term with a fixed meaning that does not require context or specification, which can contribute to confusion and imprecision. Attempts to clarify the meaning of “sex” have ranged from the rejection of any singular definition (Lehtonen and Kokko 2014) to proposing multiple different definitions (Gowaty 2013; Orive 2020; Warkentin et al. accepted). These options include references to “reproduction,” “recombination,” “meiosis,” “syngamy,” “gametes,” “anisogamy,” types of individuals, “copulation,” and more. The difficulty in specifying a singular concept to which “sex” refers reflects the dazzling array of phenotypes and processes involved in generating variation and the production of offspring (Aanen et al. 2016). The various meanings of “sex” are also a consequence of the scientific process in which scientists develop language to communicate concepts across different study systems, research questions, and socio-cultural backgrounds. To emphasise the varied contextual constructions of “sex” concepts, we place the terminology central to our discussion in quotation marks.

The conceptual multiplicity of “sex” brings about varying levels of conflict. There is little apparent tension in comprehension between using sex to mean “meiotic” “recombination” in “sexual reproduction” as opposed to phenotypes in “anisogamous” systems, even when found in close proximity (e.g., Bachtrog et al. 2014). In contrast, within specific fields the construction of what “sex” is or is not can be heated areas of debate. In particular, perspectives on “sex types” in recent literature are put into sharp opposition to each other as either a binary category based on “anisogamy” or a multivariate trait that emerges from the interaction of many factors (Goymann et al. 2023; McLaughlin et al. 2023; Smiley et al. 2024; Griffiths and Spencer 2025; Eppley et al. 2026). As with other polysemic scientific concepts with multiple meanings (e.g., species, gene, function, or fitness; Keller and Lloyd 1994; Sterner 2022), the resulting discourse over what “sex” means has been an opportunity to gain a better understanding of how constructions of “sex” have been used for different purposes corresponding to their own underlying research questions and motivations (Richardson 2022; Ritz and DuBois 2025; Watkins and DiMarco 2025; Velocci 2026). Acknowledgement of and engagement with the constructed meanings of “sex” in the sciences is particularly important because of the political and social entanglements and impacts of “sex” definitions. Scientific knowledge is shared with the broader community and scientists must contend with the ways that scientific research and communication of “sex” also participate in social and political arenas (Roughgarden 2004; Miyagi et al. 2021; Sudai et al. 2022; Lewis and Sharpe 2023; Aghi et al. 2024; Rice et al. 2025; Sedlack et al. 2026 ).

Here we propose an analytical framework of a labyrinth of “sex” as a method to reveal the ways that epistemic decisions shape the meaning of “sex” in different contexts. The structure of a labyrinth highlights that many branching paths lead to distinct destinations and different conceptual versions can arise for a specific research question based on the inclusions and possible connections among concepts. We examine the architecture of the semantic labyrinth of “sex” by considering the ways in which the meaning of associated traits can be variable and contingent. Navigating the many possibilities at each of these conceptual nodes reflects the incredible capacity of “sex” to describe and investigate the evolution of diverse traits of eukaryotic reproductive systems. Engaging with the diverse ways of understanding and implementing scientific language requires contending with the impact of terminology on the scientific process (Keller and Lloyd 1994; Hull 2001; Brigandt 2020). Thus, in the construction of concepts, scientific research includes a theoretical or philosophical enterprise influenced by cultural and historical perspectives that affect the formation of scientific knowledge (Butler 1990; Haraway 1988).

## “Gender” as an instance of constructed meanings of “sex”

The process through which the meaning of “sex” is constructed can be observed and experienced in the ongoing discussions about how to use “gender” to resolve perceived issues of “sex” in both social and scientific contexts. Versions of “gender”, proposed by sexologists, feminists, botanists, and evolutionary biologists, share a conceptual goal of providing language to identify and discuss an aspect of the variation in biological systems or lived experiences that could not be sufficiently described with “sex” alone. Consequently, the introduction of "gender" in these different contexts also redrew the ontological boundaries of "sex". There remains no singular agreement as to the right classification scheme for “gender” versus “sex,” or whether a division between these concepts is needed at all. Indeed, in many languages there continues to be no linguistic separation between “sex” and “gender", without the use of English words. The different conceptualisations, or lack thereof, of “gender” and/or “sex” across languages and cultures further emphasises that the dominant (as in Liboiron 2021) English formulation should not be assumed to be the best or only way to conceive of these properties.

The most prevalent version of “gender” is the modern English usage which is generally used to distinguish cultural aspects of a person’s social identity and behavior from their physical traits (i.e., “sex”). This version of “gender” has its roots in sexological clinics and research of the mid 20th century in which human social and psychological “sexual” variation was given a different name in order to stabilise binary concepts of physical “sex” (Hausman 1995; Fausto-Sterling 2000; Gill-Peterson 2021; Eder 2022). Subsequently feminist movements adapted this language and conceptual foundation in order to recognise and take action against patriarchal systems that structure social divisions among people based on prejudicial and changeable social circumstances. As this conceptualisation of “gender” as a trait separate from “sex” increased in prominence, scientists were encouraged to implement consistent language, specifically in delineating that the traits measured in non-human animals should strictly be the purview of “sex” (e.g., Unger 1979; Pearson 1996; Torgrimson and Minson 2005; Goymann and Brumm 2018; Richie 2019). However, the persistent conflation of “gender” and “sex” in scientific literature indicates that the distinction may not be comprehensively understood or meaningful for all biologists (Haig 2004; McLaughlin et al. 2023; SturtzSreetharan et al. 2025). The percieved misuse of “gender” in scientific research has been identified as undermining the validity of gender-diverse identities, promoting sex-essentialism, and making scientific research and education less inclusive (Casper et al. 2022; Rice et al. 2025).

In contrast to the perspective that distinctions between “sex” and “gender” will facilitate more accurate and inclusive research, the separation of these concepts has also been suggested to be an oversimplification that introduces its own biases. Notably, feminist and queer scholarship has critiqued that the conceptual separation between “gender” and “sex” reinforces perceptions of a dichotomy between “nature” and “culture” while also obscuring the ways in which “sex” is also constructed (Butler 1990; Unger and Crawford 1993; Fausto-Sterling 2000). Thus, there may not always be a clear distinction between “sex” and “gender” and language that reflects the entanglement of these concepts (e.g., “gender/sex”) may enable more accurate biomedical research in humans (Springer et al. 2012; van Anders 2015; DuBois and Shattuck-Heidorn 2021; Ritz et al. 2025). In addition, there may also be contexts in which “gender” as social identity or expression can be meaningful in animals other than humans, especially if considering behaviors in a social context or in an evolutionary framework with other hominid or primate species (Meynell and Lopez 2021; Dunsworth and Ware 2025). A strict adherence to a codified distinction between “sex” and “gender” in biological research and teaching may contribute to a loss of nuance or awareness of the contested construction of these concepts.

A different definition of “gender,” “*the appearance, behavior and life history of a sexed body*,” was proposed by eco-evolutionary biologist Joan Roughgarden in her book Evolution’s Rainbow: Diversity, Gender, and Sexuality in Nature and People (2004). This proposal distinguishes “sex” as strictly the type of “gamete” an individual produces and offers “gender” as the way to refer to the amalgamation of traits that are associated (directly or indirectly) with the individuals producing a type of “gamete” in a social context. In other words, this proposal addresses the issue that “sex” is often used to reference a conglomeration of traits by describing non-gametic phenotype components as “gender” (see also “Female,” “male” and “hermaphrodite” section). As this approach was not adopted in biological research, scientists have continued to develop new proposals for language and methods to specify the specific components of “sex” that are being studied and recognize the variability in those traits (Massa et al. 2023; McLaughlin et al. 2023; Pape et al. 2024; Smiley et al. 2024; Eppley et al. 2026). However, a resultant practice of using the word “sex” in combination with a specific trait of interest (e.g., “genetic sex” or “hormonal sex”) can contribute to inaccurate assumptions about distributions of these traits across individuals, relationships of these traits to “gamete” type production, or whether these traits can be used to determine “sex type” (see also “Sex determination and sex chromosomes” section). Indeed, if so many different components of an animal's phenotype contribute to or are “sex,” then it has been argued that the term may be effectively meaningless (Velocci 2024; Watkins and DiMarco 2025). Rougharden’s (2004) alternative definition of “gender” reverberates through the current struggle to increase clarity in research on “sex” and in further explorations into ways that “gender” can be conceptualised to describe the agential and relational properties of human and nonhuman organisms (Cuciniello, Accepted).

In seed plants “gender” has been used to refer to a quantitative property of “sex” also sometimes described as “sex expression,” “sex allocation,” or “sex role” (Pannell 2023; Lloyd 1980a). This concept has likely been elaborated in angiosperms in particular due to their modular systems in which an individual “sporophyte” plant can produce many independent “reproductive” structures of flowers that can contain "stamen" with "ovules" and/or “pistils” with “pollen” (Cronk 2022a; Subramaniam and Bartlett 2023). “Ovules” and "pollen" are often labeled as “female” or “male,” respectively, as they contain either mega or micro “gametophytes” that subsequently produce “dispersed” or “retained” “gametes.” Functional flowering plant “gender” is not only a description of the type of “gamete” production of an individual (i.e., phenotypic “gender” with “reproductive” structures or flowers often measured as a proxy) but also the likelihood of genetic contribution to the next generation through a particular “gamete” type (Pannell 2023; Lloyd 1980a). For example, a “self-incompatible” “hermaphroditic” individual surrounded only by “pollen”-producing plants will have a quantitative measure of “gender” the same as that of a “female” “dioecious” plant (i.e., reproduce only through “gametes” produced in “ovules”), whereas the same individual surrounded only by “ovule”-producing plants will reproduce only through “pollen” and have a value of “gender” that is the same as a “male” “dioecious” plant. Notably, these context-dependent measurements of functional plant “gender” can have more meaningful implications for identifying how selection can influence “sexual system” evolution than measures of phenotypic “gender” (Charlesworth and Charlesworth 1981; Charnov 1982; Delph and Wolf 2005; Lloyd 1980b). Although not specifically included in the original formulation, concepts of angiosperm “gender” may also be useful for more accurately describing “sexual” properties of all land plants and even hermaphroditic animals (DeWitt 1996; Jesson and Garnock-Jones 2012). Probing plant “gender” reveals the insufficiencies of the available language to fully describe “sex” in non-animal taxa as well as the sensitivity and awareness needed in the interface between scientific language and society (Oberle and Fairchild 2023; Pannell 2023; Subramaniam and Bartlett 2023; McCarren et al. 2025 ).

The fluidity in the meaning of “gender” is illustrated by the alternative definitions across time and taxa. Can these multiple definitions persist independently? Can learning across these disciplinary divisions provide greater insight into what “gender” can represent (Dudle and Altman 2006)? Or will the associations of “gender” in the human social context always bring culturally laden meanings that impact and potentially bias research and contribute to marginalization of gender, sex, and sexuality diverse individuals (Oberle and Fairchild 2023; Rice et al. 2025; McCarren et al. 2025 )? There is not necessarily one right answer to these questions. Rather, from each usage of “gender,” we learn about the many different traits scientists and scholars want to investigate and their research perspectives. Understanding “gender” as part of the labyrinth of “sex” provides an opportunity to identify the ways in which conceptual choices shape the meaning of “sex” and subsequently influence the structure of knowledge on biological phenomena.

## The semantic labyrinth of “sex”

Although any particular meaning of “sex” may be appropriate for a specific question or taxon, each formulation is embedded within a specific perspective and applicability beyond that context cannot be taken for granted (Richardson 2022). Approaching “sex” instead as a compound of many different concepts allows for the different meanings of “sex” to be understood as reflections of alternate paths through a labyrinth of connections. Navigating the labyrinth of “sex” requires confronting each of the component traits of “sex” and considering how their meaning can be variable across different contexts with compounding contingencies reflecting their evolutionary relationships and conceptual interconnection (Table 1; see also Warkentin et al. accepted).

**Table 1:** Variable and contingent meanings of traits often associated with “sex.”

“Sex” traits	Variability and contingency in meaning
**“Reproduction”**	No consistent association between "reproductive" mode (i.e., “asexual” and “sexual”) and presence of “recombination,” “gametes,” or “syngamy”. Different modes of “reproduction” can co-occur within an individual and/or lineage "Sexual reproduction” is a conflation of two distinct processes, “meiosis” and “syngamy” The process of “sexual reproduction” can span multiple generations
**“Recombination”**	Can occur independently of “reproduction” Can include incorporation of genomic material from a different species Can occur across different types of “reproduction,” including “meiosis” in “sexual reproduction” as well as “asyngamous” recombination in “gametes” with altered “meiosis” (e.g., “parthenogenesis”), or in non-“meiotic” “reproduction” (e.g., “parasexual”)
**“Meiosis”**	Can result in haploid cells that can be directly involved in “syngamy” and “reproduction” (e.g., “gametes”) or that develop into haploid individuals (e.g., “spores”) Variation in the mechanisms of "meiotic" division can contribute to differences in number and size of "meiotic" products as well as the extent of “recombination”
**“Syngamy”**	Does not only occur between single, independent, haploid cells (e.g., “gametes”) . Cell fusion (“plasmogamy”) is not always followed rapidly by nuclear fusion (“karyogamy”) Can occur multiple times before the completion of a “sexual reproductive” life cycle Nuclear fusion may be absent and the nuclear material of one cell may be excluded (e.g., “pseudogamy”)
**“Gamete”**	Can be a product of “meiosis”, mitosis after “meiosis”, or altered “meiosis”. Not all “gametes” are capable of "fertilisation" or require “syngamy” to contribute to “reproduction” (e.g.,“parthenogenesis”) Within lineage variation in morphology and/or genotype may influence "gametic" compatibility for “syngamy” and “reproduction” There are multiple independent origins of differently sized morphologies of “gametes” with disassortative fusion (“anisogamy”) Anisogamous “gamete” pairs may vary in the extent of differences including morphology, cytoplasmic inheritance, cost to produce, or mobility, among other traits Anisogamous “gametes” are often described as “female” (relatively larger “gamete”) or “male” (relatively smaller “gamete”) referencing the cultural labels of organisms with which these “gametes” were historically associated Anisogamous “gamete” pairs may vary in the extent of differences including morphology, cytoplasmic inheritance, cost to produce, or mobility, among other traits
**“Female”** and “**Male”**	Have historical and cultural associations that predate and influence their scientific usage Applied to individuals, life stages, and/or traits across different stages of the life cycle Defined by different traits depending on the lineages and context, which may or may not have a direct relationship to the morphology of “anisogamous” “gamete(s)” produced Can correspond to a range of genetic variation from distinct karyotypes (e.g., “sex chromosomes”) to no consistent genetic differences Can include multiple stable phenotypes that produce the same type of “gamete(s)” May correspond to phenotypic expression of “gametes” but not reflect additional factors that influence “reproductive” compatibility and outcomes (e.g., “sexual system”) Have also been used to describe individuals in lineages with “asexual reproduction” (e.g., “parthenogenesis”) Have also been used to describe categories in lineages without “gametes” and/or “anisogamy”
**“Sex chromosome”**	Many mechanisms of influence on phenotypic development, not only the presence/absence of a single gene Influence on phenotypic development can be overridden by other factors (e.g., “sex reversal”) Can be varying levels of “recombination”, arrangement, number, and extent of differentiation “Sex chromosome” systems can rapidly turn over
**“Sexual system”**	Naming varies with respect to organismal differences in lifecycle and phenotypes associated with “gamete” production (e.g., “female” and “male”) which may include phenotypes that are rare or difficult to identify. “Reproduction” among individuals in a system can be influenced by “gametic” or genetic compatibility for “syngamy” as well as traits that influence individual phenotypic compatibility (e.g., “self-compatibility”) which may include “gamete” production phenotype (e.g., “female” and “male”) “Reproduction” can be influenced by more than the "gamete" producing individuals, including multispecies interactions

Notes: We describe some of the variabilities in the meaning of each concept, especially those that contravene typically assumed properties of that concept. Concepts are ordered corresponding to the progression of the main text, which includes further details.

### “A/sexual reproduction”

#### Inconsistent criteria distinguish “asexual” and “sexual” types of “reproduction”

“Reproduction” relates to the formation of new individuals and is an essential component of the perpetuation of a lineage (but see Griesemer 2016). The term “sexual reproduction” is generally associated with a combination of processes found in most eukaryotic species that can generate genetically distinct individuals through “meiosis” (ploidy reduction with “recombination” and chromosomal segregation) followed by “syngamy” (ploidy restoration with genetic exchange) (Jeffries et al. 2025; Roze et al. 2025). “Asexual reproduction,” in contrast, serves as an umbrella term for many different forms of “reproduction,” united solely by their departure from “sexual reproduction.” This category can include types of “reproduction” absent of virtually everything associated with “sexual reproduction” (“agametic” or “vegetative”) as well as alterations during or before “meiosis” (“parthenogenesis”) and even the “reproduction” of “clones” from a different species (“xenoparity”; Orive and Krueger-Hadfield 2021; Juvé et al. 2025). Distinct but related issues of “sexual” bias in scientific thinking include how common biological concepts such as “individual” (e.g., Folse and Roughgarden 2010; Herron et al. 2013) and “species” (e.g., Hörandl 2018) are often constructed with respect to obligate “sexually reproducing” organisms and may not meaningfully apply to lineages with obligate or facultative “asexual reproduction.” In addition, persistent assumptions about organisms with “asexual reproduction” can be oversimplifications, such as that they feature less genetic variation (Gorelick and Heng 2011), innovation (Tripp 2016), and diversity (Felsenstein 1981), reflecting a historical underappreciation and lack of understanding for “asexual reproduction” (Boyden 1954). Instances of conceptual disjunction such as these can present critical opportunities to reflect on the ways that bias towards more familiar systems can influence scientific thinking and develop alternatives (Kokko 2017; Hörandl 2018). By interrogating the different proposals for what is and is not “sexual,” we can appreciate that modes of “reproduction” do not fit into discrete binary categories and make more nuanced comparisons on the evolutionary impacts of “sexual” processes (Jeffries et al. 2025).

#### “Recombination” and genetic exchange are not restricted to canonical “sexual reproduction”

The “sex” in “sexual reproduction” can be interpreted to describe how the processes of “meiosis” and “syngamy” introduce genetic variation during “reproduction.” However, the restriction of this aspect of “sex” to “sexual reproduction” omits the many alternate mechanisms through which genetic variation can be generated in individuals or their offspring. Thus, Redfield (2001) argued that “sex” should be instead defined more broadly to include any form of genetic exchange and “recombination.” This conceptual change would classify phenomena common in bacteria and archaea such as conjugation, transformation, and transduction, not all of which involve “reproduction,” as “sex” or “sexual” processes (Smith et al. 1991; Redfield 2001; Otto and Lenormand 2002; Narra and Ochman 2006; Moradigaravand and Engelstädter 2013; Ambur et al. 2016). This more inclusive definition of “sex” could lend to greater appreciation of the high rates of horizontal gene transfer and mitotic “recombination” found in the vast diversity of eukaryotes (Tilquin and Kokko 2016; Van Etten and Bhattacharya 2020; Bulankova et al. 2021), multiple mechanisms of genomic variation within organisms (Wilson et al. 2024), and facilitate comparisons across multiple independent origins to understand why “sex” (i.e., genetic exchange and “recombination”) has evolved (Redfield 2001). The counterargument is that precise language that differentiates mechanisms would better facilitate mutual understanding and comparison of specific traits (Martinez 1991). For instance, the terminology of “parasex” to describe a form of “reproduction” in fungi and unicellular organisms has been effective in highlighting the similar outcomes (i.e., production of genetically distinct offspring) and processes (i.e., ploidy cycling, genetic exchange, “recombination”) as “sex” while reflecting the absence of “meiosis” (Kondrashov 1994; Loidl 2016; Yadav et al. 2023). These different proposals for what constitutes “sexual” events or qualities demonstrates how any specific concept is enmeshed in a multitude of processes (Lehtonen et al. 2013).

#### “Sexual reproduction” is a conflation of two distinct processes, “meiosis” and “syngamy” that does not necessarily involve a "gamete"

“Sexual reproduction” as a eukaryotic trait of “meiosis” and “syngamy” fuses together two distinct processes, characterised by John Maynard Smith (quoted in Hull 2001) as cell division (“reproduction”) and fusion (“sex”). Although these two processes are closely linked in canonical (animal) reproduction, they can also occur independently which lends to challenges in specifying when in the life cycle “sex” occurs and what transitions count as “sex” in other eukaryotic organisms (Cronk 2022a; Gorelick and Carpinone 2009; Griesemer 2016; Jeffries et al. 2025). For example, in plants and fungi, the presence of both haploid and diploid life phases does not fit with constructions of the animal “unitary” individual, with consequences for how haploid selection and “self-fertilization” are conceptualised (Cronk 2022a; Boudouresque 2015; Umen and Coelho 2019). In these life cycles, it is unclear whether transition to a haploid lifestage could be considered “sex” because it involves “meiosis,” or if “sex” is a multi-generational trait that is only completed following “syngamy.” Basidiomycete fungi further contravene “canonical” expectations of when “sex” occurs because following cellular fusion (“plasmogamy”) they form a stable dikaryotic state without nuclear fusion and completion of “syngamy” (Young et al. in preparation; Casselton and Olesnicky 1998). A dikaryotic individual can even “mate” again, fusing and exchanging nuclei with a monokaryon (Anderson and Kohn 2014). Life cycles with both reduced and unreduced stages requires considering whether multiple stages can have “sexual identities” or whether “sexual” attributes are “transferred” to the generation that produces the cells that undergo “syngamy” and complete the sexual cycle (see also *“Female,” “male,” and “hermaphrodite”* section; Cronk 2022a; Meissner 2021). Taking the “sexual reproductive” cycle into account can lend to a different orientation toward the recognition of relationships among “sexual” structures, “meiotic” products, and cells that participate in “syngamy” (Bai 2015).

The synonymization of “sexual reproduction” with “meiosis” in particular can be seen in the term “meiotic sex” (Beukeboom and Perrin 2014) as well as in the interpretation of intact “meiotic” genes as evidence for “sexual reproduction” in a lineage (Schurko and Logsdon 2008). As definitive as this single trait may seem for establishing “sexual reproduction,” “meiosis” can be quite variable. For instance, the outcome for “meiotic” products can range from unicellular organisms, to spores that develop into independent haploid life stages, to specialised “sexual” cells. The function of “meiotic” cells can even differ in the same individual or population, with some cells requiring syngamy to develop and some not, such as in facultative parthenogenesis or “haplodiploid” species. In addition, “meiosis” can result in different numbers and sizes of haploid cells and have different mechanisms of homologous chromosome pairing, “recombination,” separation, and reduction (Gorelick et al. 2016; Loidl 2016).

The different outcomes for “meiotic” cells, especially outside of the animal kingdom, necessitates interrogating the concept of a “sexual” cell or “gamete” because this term is not synonymous with the product of “meiosis”; not all “gametes” are capable of “fertilisation,” not all cells that engage in fusion are single haploid cells, and not all “sexual” life-cycles have easily identifiable “gametes” (see also *“Female,” “male,” and “hermaphrodite”* section). A “gamete” also cannot be defined based on a contribution to “sexual” reproduction as the term is applied even in cases in which meiotic products have altered morphology, or even no nuclear material, and never engage in syngamy (e.g., “sperm” heteromorphism; Till-Bottraud et al. 2005) or in which syngamy results in the development of a nutritive tissue instead of an embryo (“double fertilisation”; Dresselhaus et al. 2016; Li and Yang 2020). “Sexual reproduction” can even involve more than only two “gametes” such as in systems that are permissive of “polyspermy” which can result in chimeric individuals that consist of different parental genomes and/or ploidies (Schwander and Oldroyd 2016; Aamidor et al. 2018). Nevertheless, distinguishing forms of “reproduction” based on the involvement of a “gamete” can be informative, especially among different forms of “asexual reproduction” as systems that involve “gametes” have marked differences in how mutations are inherited by offspring compared to “agametic” processes (Boyden 1950; Orive and Krueger-Hadfield 2021). Describing “reproductive” modes with an emphasis on relationships between the genotype of the “gametes” and offspring can facilitate more comprehensive comparisons across taxa compared to the simplistic categories of “sexual” and “asexual” (see also *Systems of “a/sexual” individuals* section; Curtis 1900; Yadav et al. 2023).

#### Derived “asexual reproduction” or “parthenogenesis” does not imply the absence of “recombination” or “syngamy”

Modifications to “meiosis” and “syngamy” can be as complex and diverse as the processes from which they originated and are categorized as derived “asexual reproduction” or “parthenogenesis” once they are deemed sufficiently altered. However, different perspectives on the criteria necessary for “reproduction” to be “sexual” can contribute to inconsistent designations into categories which provide incomplete information about inheritance patterns across mechanisms (Lehtonen et al. 2013). For example, identifying mechanisms that limit “recombination” and bias parental genome transmission, such as in both “paternal genome elimination” and “hybridogenesis,” can have more comparative utility than merely delineating whether or not the system is classified as “sexual” (Lehtonen et al. 2013; Lavanchy and Schwander 2019; Herbette and Ross 2023). This could be achieved by measuring the impact of different systems on the asymmetry of inheritance in a multidimensional space including variables such as parental transmission bias and bias in the resulting diploid genome (Ross et al. 2022). Distinctions between “sexual” and “asexual” may also not apply equally well across taxa, such as the similar outcomes found in “homothallic” “sexual reproduction” of haploid organisms in which intratetrad “selfing” can occur between cells produced by a single round of “meiosis,” and "asexual" "parthenogenesis" in intratetrad fusion or aborted division during “meiosis” in plants and animals (see also *Systems of “a/sexual” individuals* section; Hood and Antonovics 2004; Billiard et al. 2012). Furthermore, forms of “sexual” “selfing” can result in even less genetic variation than forms of “asexual reproduction,” such as in haploid “intragametophytic selfing” between mitotic “gametes” of the same “meiotic” product (Cronk 2022a; Haig 2016). Thus, common associations between “reproductive mode” and genetic variation in the next generation are overly simplistic, as forms of derived “asexual reproduction” can involve “asyngamous” “recombination” while “sexual reproduction” can feature severely limited “recombination” or very little introduction of genetic variation (Lenormand et al. 2016; Engelstädter 2017; Jaron et al. 2021; Wilson et al. 2024).

The inconsistent terminology used for derived “asexual reproduction” within and across major eukaryotic groups amplifies confusion for the criteria associated with this category (Neiman et al. 2014). For example, “parthenogenesis” does not refer to a consistent mechanism or provide information about the presence or absence of “meiosis” across plant and animal kingdoms (Neiman et al. 2014). In addition, the implication of “parthenogenesis” as “reproduction” by a single individual is not consistent with the necessary behavioral or “gametic” (“pseudogamous”) interactions among individuals of the same or different lineages (Beukeboom and Vrijenhoek 1998; Neiman 2004; Lehtonen et al. 2013). In systems with ancestral “anisogamy,” “parthenogenesis” is often assumed to occur only in “female” organisms as the larger “oocyte” is considered necessary to provide cytoplasmic material to offspring (see *“Female,” “male,” and “hermaphrodite”* section). However, rare cases of all “male” lineages in “egg”-dependent “androgenesis” have been observed (which can even involve fusion of two “sperm” within the “egg”) as well as “sperm”-dependent “gynogenesis” in all “female” lineages (McKone and Halpern 2003; Lehtonen et al. 2013; Schwander and Oldroyd 2016; Aamidor et al. 2018). Retention of “sexed” labels and language in reference to“asexual” organisms alters the associations of “sex types” to focus on phylogenetic relationships to “gamete” production rather than meanings associated with a specific form of “sexual reproduction” (Franklin-Hall 2020). Notably, the former construction is useful as it can facilitate comparisons among closely related species on the impacts of “reproductive” mode on phenotypic evolution (Burns and Tsurusaki 2016; Huylmans et al. 2021; Djordjevic et al. 2025).

Additional concerns with the term “parthenogenesis” have been raised throughout its use, including that the implication of “virgin” in the greek root is not an accurate reflection of the potential “mated” state of individuals nor does it apply to systems with “external fertilisation” (Lankester 1917; Mittwoch 1978). In addition, social stereotypes of “celibacy,” and “femininity” contribute to biased language used to describe “parthenogenetic” animals (Ebeling 2006). Types of asexual reproduction involving multiple individuals (i.e., gynogenesis) have also been subject to biased language in descriptions of “sexual parasitism” or “sperm stealing” (Ebeling 2006). References to derived “asexual” lineages as “unisexual” can be a source of confusion in communication across taxa, as “unisexual” is also used in “isogamous” fungal systems to describe “sexual reproduction” between individuals of the same “mating type” (Wilson et al. 2015; Sun et al. 2025). Despite these discrepancies in language, investigations into the genetic, genomic, and developmental phenomena that drive transitions among clearly distinct “reproductive” modes provide critical insights into the mechanistic basis of “reproduction” and evolutionary maintenance of “meiosis” and “syngamy” (Neiman and Schwander 2011; Neiman et al. 2018).

### “Female,” “male,” and “hermaphrodite”

#### “Female” and “male” have many different definitions and implications

Perhaps the most familiar use of the term “sex” is in the categorisation of components of “sexual systems” as either “female” or “male.” These terms reflect pre-existing social categories that are imbued with historical and cultural context (Martin 1991; Ahnesjö et al. 2020; Rosenthal and Ryan 2022; Subramaniam and Bartlett 2023; Velocci 2026). As a result, the influence of preconceived biases associated with these categories is evident across biological research, including in “fertilisation” (The Biology and Gender Study Group et al. 1988; Martin 1991), “genital” evolution (Ah-King et al. 2014), and “sexual selection” (Hrdy 1986; Gowaty 1997; Gowaty 2003; Tang-Martinez and Ryder 2005; Tang-Martínez 2016; Ah-King 2022). In particular, the “sex role” concept has been critiqued for contributing to the construction of stereotypical and anthropomorphic expectations for “female” and “male” animals (Ah-King and Ahnesjö 2013). Nevertheless, these categories have persisted in biological research likely due to both the history of their use in structuring scientific thinking and their correlation with phenotypic variation and mechanisms of reproduction in overrepresented animal study systems.

The meaning of “male” and “female” and how they are identified is rarely specified in scientific studies. The different traits used to assign individuals into “sex type” categories and the different levels of biological organisation in which “sex types” are considered (e.g., cells, tissues, lifestages, and/or individuals) contributes to formulations of “sex” that are stretched to include even cells in culture which may have long been isolated from the properties that were initially associated with the “sexed” category (Ritz 2017; Richardson 2022: Pape et al. 2024). Recognition that “sex types” often describe many different traits within individuals has also led to increasing formulations of a continuous spectrum of "sex" or multivariate “sex” that defies binary categorisation (Massa et al. 2023; McLaughlin et al. 2023; Smiley et al. 2024). These critiques and re-formulations of “sex types” have been incredibly impactful in encouraging scientists to specify the actual traits they are using to categorise individuals, consider the causal factors that are related to traits of interest, and structure research to more accurately investigate phenotypic variation (Thinius and Trappes 2024; Pape et al. 2024; Smiley et al. 2024; Eppely et al. 2026). The issues of "sex" these different perspectives aim to ammeliorate have also been identified as reasons to dispence of "sex types" concepts (Velocci 2024; Watkins and DiMarco 2025). In contrast, there have also been arguments for the enactment of a singular definition of “sex types” in relation to “anisogamy” (different sized “gametes” with disassortative fusion) in which “female” is associated with the larger “gamete” and “male” is associated with the smaller “gamete” (Goymann et al. 2023; Griffiths and Spencer 2025). In this approach no other traits should be assumed to correspond to “sex types” and phenotypic variation at the organismal level, including those traits often associated with “sex types,” would be described with a different term (see also *“Gender” as an instance of constructed meanings of “sex”* section). Disagreements in the literature reflect not only differences in perspective on how “sex types” are or should be understood in biology but also different frameworks of “sex types” that are useful for a specific research question (i.e., a category, trait, or dynamic system; Ritz and DuBois 2025).

The explanatory uses of “sex types” in biological research can be summarised as *“who you* [an individual] *are, who you can mate with, and who your parents are”* (Whitfield 2004). The “anisogamy” definition of “sex types,” frequently implemented in the evolution of “sex” research (e.g., Bachtrog et al. 2014; Beukeboom and Perrin 2014), can capture these properties in some systems because gamete compatibility and morphology are linked to histories of selection on distinct developmental lineages (Franklin Hall 2020). However, patterns of “gamete” production alone are inadequate to fully (1) describe the variation in phenotypes which may, or may not be, associated with “gamete” production or variation in allocation to different types of “gamete” production within an individual, (2) indicate which individuals can “reproduce” together, or (3) reflect the genetic relationship or ploidy of the “parents” that produced the “gametes” (see also *Systems of “a/sexual individuals* section). Anisogamy based “sex type” categories alone provide limited insight into the intra- and interorganismal interactions which influence the evolution of “reproductive” characters as well as the ecological factors that contribute to the variation in "reproductive"/"sexual" strategies (Cortés-García et al. 2024; Gourevitch et al. 2026).

#### “Female” and “male” are not sufficient to describe phenotypic variation within a lineage

The continuous variation in traits associated with “sex types” such as karyotype, genotype, gene expression, morphology, hormones, or behavior, and their inconsistent correlation, hinders the use of traits associated with “sex” as defining features of “sex types” especially when comparing across different taxa (Massa et al. 2023; McLaughlin et al. 2023; Pape et al. 2024; Smiley et al. 2024, Eppley et al. 2026). The amorphous category of “intersex,” demonstrates the limited utility of dimorphic categories to reflect phenotypic variation in the development of traits associated with “sex” (Fausto-Sterling 2000; Roughgarden 2004; Velocci 2026). For instance, depending on the definition, “gynandromorph” can describe individuals that have a mixture of features associated with both “females” and “males” as a result of different genotypes (e.g., in systems with polyspermy) or ploidies, endosymbiont presence in the cytoplasm, and/or mutations in genes associated with “sexual development” (Pereira et al. 2010; Schwander and Oldroyd 2016; Fusco and Minelli 2023). Research on “sex types,” in particular, must contend with how, in the process of scientific research, the tremendous variation present in nature has been oversimplified into discrete categories constructed within patriarchal and eugenic social contexts (Velocci 2026). Challenging constructions of “normal” embedded in the language and conceptions of “sex” can help confront facile categories and contribute to a more accurate representation of the variation in “sexual” traits (Fausto-Sterling 2000; Packer and Lambert 2022; Lewis and Sharpe 2023; Velocci 2026).

A dichotomy of “sex types” is also too limiting to describe the incredible variation in phenotypes among individuals that produce the same type of “gamete” in a lineage. An extreme example of phenotypic variation within a “sex type” can be found in eusocial organisms, which can have both “reproductive” and non-“reproductive” phenotypes (Ross et al. 2013). There can also be “sexual polymorphisms,” that constitute distinct phenotypes specific to one “sex type” (Mank 2023). These polymorphisms may include “alternative reproductive tactics” (Oliveira et al. 2008), which are often described by culturally embedded stereotypes such as “bourgeois males” that defend territories versus smaller “parasitic males” that may “sneak” “mating” opportunities (Taborsky 1997; Roughgarden 2004). Descriptions of “alternative reproductive tactics” also tend to be focused on “males” even though they can also be found in “females” (Wang et al. 2024). As “alternative reproductive tactics” represent different types of “reproductive” strategies, Brockmann (2001) proposed that they can be considered analogous to “sex types” and categorized as either fixed (akin to “gonochorism”) or reversible/facultative (akin to “hermaphroditism”). Moreover, “sexual polymorphisms” may be maintained by disruptive selection to resolve “intralocus tactical conflict,” similar to mechanisms of “intersexual conflict” associated with phenotypic variation among “sex types” (Morris et al. 2013; Abbott et al. 2019). Together, these examples point towards the ways that scientists already conceive of “sex types” as polymorphisms with multiple possible “sexual” varieties and the need for language of “sex types” that facilitates investigations into their evolutionary origins.

#### “Hermaphrodite” contains many variations in the spatial and temporal expression of anisogamous “gametes” production

“Hermaphrodites” (individuals that produce, or have the “potential” to produce, both types of “anisogamous” “gametes”) further test the limits of “sex type” terminology (Schärer 2017). Notably, not all individuals that appear to produce both types of “gametes” are necessarily “hermaphrodites.” For example, some insects often classified as simultaneous “hermaphrodites" instead appear to possess a distinct, transgenerationally inherited “sperm” cell lineage in an otherwise phenotypically “female” background (Royer 1975; Mongue et al. 2021). Conversely, the label “hermaphrodite” may apply to individuals that do not obviously produce more than one type of “gamete” or even “gametes” at all if they constitute a genetic individual whose modular parts or colonies produce different types of “gametes” (Cronk 2022a; Cox 1990; Wasson and Newberry 1997). In this case, “haplodiploid” lineages could be creatively described as cross-generational “hermaphroditism” in which the genetic individual of the “queen” includes both herself as the “female” and the haploid “clonal” “male.” Even for lineages in which individuals are directly observed to produce both “gamete” types, whether “hermaphrodite” constitutes a “sex type” is controversial. Nevertheless, references to “three sexes” in species with “trioecy” (i.e., “females,” “males,” and “hermaphrodites” in the population) supports the interpretation that “hermaphrodite” is being used in the scientific literature to describe a type of “sex” (e.g., Kaliszewicz and Lipińska 2013; Tandonnet et al. 2019; Roy 2021; Wang et al. 2025).

The phenotype of “hermaphrodite” does not necessarily correspond to an individual’s allocation into and/or “reproductive” success through both of the different types of “gametes” they produce. A quantitative measurement, such as plant “gender,” to describe the likely functional realisation of an organism’s “gamete” production may be more informative than the broad category of “hermaphrodite” (see also *“Gender” as an instance of constructed meanings of “sex”*; Pannell 2023; Lloyd 1980a). However, even the additional nuance captured in this measure does not account for all the factors that can influence “reproductive” outcomes, such as potential differences in “quality” or “specialisation” between “reproductive” functions (Robbins and Travis 1986). Descriptions of facultative variation in the “sex roles” of a “hermaphroditic” individual convey how the phenotypic expression of both “gamete” types can result in different “reproductive” outcomes between individuals or even different “sexual” interactions (Koene and Ter Maat 2007; Nakadera et al. 2015; Koene 2017). Conceptualising and communicating attributes of a “hermaphroditic” individual reveals the disconnection in using the same terms (i.e., “female” and “male”) to describe both a “sex type” of an individual as well as component parts of a “hermaphroditic” individual. Notably, the use of “male” and “female” in sequentially “hermaphroditic” individuals can contribute to confusion over whether the “sex type” label is a description of a trait (i.e., current state of an organism) or a category that reflects their lifetime potential of “gamete” production.

Even the inclusion of “hermaphrodite” as a “sex type” would not be sufficient by itself to describe the many ways that individuals can produce multiple types of “gametes” with different impacts on opportunities for “sexual reproduction.” In crustaceans, for example, six types or variants of “males,” three of “females,” and two of “hermaphrodites” have been described (Benvenuto and Weeks 2020). This additional language is necessary to describe attributes of systems such as “protandric” simultaneous “hermaphroditism” (or “adolescent protandry”) in which there is an initial “male” developmental stage followed by simultaneous “hermaphroditism” (Baeza 2007; Baeza 2018). Terminology in angiosperm plants contains even more nuanced language corresponding to their modular organization, in which the “sex” of each “flower” (“perfect”/“bisexual”/”hermaphroditic,” “female,” or “male”) can be found in every possible configuration to generate an array of “sex types” at the level of the plant, including at least five different “hermaphroditic” types (e.g., “monoclinous” with only “bisexual flowers,” “monoecious” with “female” and “male” flowers, “andromonoecious” with “male” and “bisexual” flowers, “gynomonoecious” with “female” and “bisexual” flowers, and “trioecious” with all types of flowers) and potentially even more with respect to relative expression of different “flowers” (Korpelainen 1998; Cronk 2022b). In addition, terminology in bryophytes further specifies “hermaphroditic” “sex types” based on the spatial arrangement and clustering of “reproductive” structures (i.e., “gametangia”; Wyatt 1985). Although there have been repeated arguments for the efficacy of a shared vocabulary for plant “sexual types,” differences in language have persisted and are accompanied by disagreements in the levels of biological organisation to which the terms “female” and “male” should be applied and whether they should be used at all (Thieret 1973; Wagner 1975; Cruden and Lloyd 1995; Subramaniam and Bartlett 2023). Regardless of the name, recognising lineages in which individuals produce more than one type of “gamete” across taxa can facilitate comparisons into the similarities and differences in phenomena such as “sex change” (Policansky 1982; Vega-Frutis et al. 2014). The evolutionary relevance of the many different forms of “hermaphroditism” can be seen in their influence on which individuals can “reproduce” together, yet these impacts are often not included or acknowledged in simple binary classifications of “sex types” (see also *Systems of “a/sexual individuals* section; Pannell and Jordan 2022).

#### Disassortative fusion between different types of “gametes” is only one factor that influences “reproductive” compatibility

“Reproductive” compatibility is implied in an “anisogamy” definition of “sex types” (i.e., *disassortative* fusion between differently sized types of “gametes”), and is often considered, especially in animal research, one of the main factors that can influence which individuals reproduce together (see also *Systems of “a/sexual” individuals* section). However, especially in “hermaphrodites,” genetic (e.g., “self-sterility”) or phenotypic (e.g., temporal or spatial separation in “gamete” production or availability) factors that influence the potential for “self-fertilization” can have more of an impact on “reproductive” compatability within and among individuals than “gamete” types (Barrett 2002; Jarne and Auld 2006). In some cases, mechanisms to prevent “self-fertilization” can further generate “sexual” polymorphisms in the population such as in “heterodichogamy,” in which the different timing of “flower” opening among plants (e.g., “male” flowers in the morning and “female” flowers in the afternoon, or vice versa) determines which individuals are able to “reproduce” together (Endress 2020). Although less common than in “hermaphroditic” systems, compatibility can also structure dynamics of “reproduction” in systems in which individuals produce only one type of “gamete.” For instance, a chromosomal inversion in the white-throated sparrow has led to disassortative “reproduction” between different morphs often referred to as “four sexes” (Tuttle et al. 2016). In addition, endosymbionts, such as *Wolbachia*, can influence "reproductive" success among “sex types” through cytoplasmic incompatibility which can prevent “reproduction” among individuals that are not both “infected” with the same strain (Pontier and Schweisguth 2015; Hochstrasser 2023). Incorporating compatibility into “anisogamous” “sex types” also facilitates a direct comparison to “mating types” in “isogamous” species without applying “sex types” to fungi (Young et al. in preparation; Young and Pringle 2026). This framework has been used to investigate patterns across eukaryotes of why lineages with two “reproductively” compatible types evolve more frequently than those with three or more (Billiard et al. 2011; Lehtonen et al. 2016; Constable and Kokko 2018; James 2023). The influence of compatibility on how individuals can contribute to the next generation supports the importance of having language to identify and describe these different types of individuals and systems (see *Systems of “a/sexual” individuals* section).

#### “Female” and “male” are used across independent origins of “anisogamy” and even in organisms without “anisogamy”

Using “female” and “male” across all independent origins of “anisogamy” may obscure that other than the *relative* size of “gametes,” there are no universal differences between “gamete” types or individuals producing those “gametes” (Gorelick et al. 2013; Gorelick et al. 2016). The label of “gamete” is not specific and can include haploid cells that undergo “syngamy” as part of “sexual reproduction” whether they are the product of meiosis or mitosis as well as meiotic cells that do not contribute to reproduction or cells with altered meiosis and derived “asexual reproduction” (see also *“A/Sexual" reproduction* section). Diversity among “gametes” reflects the incredible variation in “meiotic” processes with striking disparities in size and structure found in both “male” “sperm” and “female” “oocytes” across taxa (e.g., Church et al. 2019; Kahrl et al. 2021). Notably, there are no consistent relationships across all organisms with “anisogamy” between “gamete” size and cost or energy expenditure in "gamete production", number of "gametes" produced, or properties such as motility or cytoplasmic inheritance (Gorelick et al. 2013). The concerns of binary “sex type” language are further exacerbated when these categories are applied to species without “anisogamy,” or even without clearly differentiated “gametic” cell types. Recent perspectives have emphasized the value of taxon-specific language to increase the accurate representation of “reproductive systems,” especially in plant and fungal lineages (Young et al. in preparation; Meissner 2021; Sandford 2022; Subramaniam and Bartlett 2023). One benefit of taxon-specific language is that it allows facets of “reproduction” to be considered on their own terms, without the assumptions that burden many animal-centric formulations of “sex types.” More generally, the development of “sex”-neutral language can promote, rather than preclude, accurate and unbiased comparisons across kingdoms (Gowaty 1997; Gowaty 2013; Ah-King and Gowaty 2015; Rosenthal and Ryan 2022; Subramaniam and Bartlett 2023; Evron 2024; Sandell et al. 2025). However, the introduction of new, or modification of existing, language must also be balanced by recognizing the utility of broad terminology like “female” and “male” to facilitate comparing similar phenomena across taxa, such as the evolution of genotypes or phenotypes across independent origins of “sex types.”

### “Sex determination” and “sex chromosomes”

#### “Sex determination” can refer to different properties, depending on the context of “reproduction,” “sex types,” and “sexual systems”

With the formulation of different concepts of “sex,” research can investigate how and why variations in "sex" arise and are maintained within and between lineages. The specific meaning of “sex determination” is influenced by how “sex” is being formulated in that particular context. For example, “sex determination” could be used to describe the factors that influence facultative “asexual” versus “sexual” reproduction (Korpelainen 1990). More commonly, “sex determination” is used in reference to how an individual develops into a particular “sex type,” often representing phenotypic categories with a relationship to “sexual” reproduction (but see *“Female,” “male,” and “hermaphrodite”* section). However the array of meanings and associations of “sex types” across different life cycles and “sexual systems” can contribute to context-specific implications of “sex determination.” For instance, In “haplodiploid” organisms, the mechanism of “sex determination” is nested within differences in ploidy or stages of the “sexual reproductive” cycle. “Sex determination” can also have different connotations depending on whether “sex types” are fixed or variable (i.e., in “hermaphroditic” “sex change”). In addition, if “sex types” refers to aspects of alternative phenotypes or “sexual” compatibility among individuals, “sex determination” can also be used in those contexts. The implications of “sex determination” changes as the meaning of “sex” shifts between being a category versus a trait of an individual, further influenced by differences across life cycles and explanatory perspective of research questions.

#### Disciplinary differences and contested boundaries between “sex determination” and “sex differentiation”

From a developmental biology perspective (primarily from research in animals), “sex determination” is understood as the process of commitment to a “sexual” fate (i.e., phenotype associated with a “sex type”) by cells and organs at multiple levels of biological organization whereas “sex differentiation" is the developmental realisation of that fate (Wilhelm et al. 2025). For example, early in ontogeny many animals have a bipotential primordial “gonad” and “sex determination” consists of the accumulation of molecular changes until a threshold is crossed and the direction of differentiation is stabilized towards either “ovaries” or “testes” (Capel 2000; Capel 2017; Weber and Capel 2021). There is vast taxonomic variation in the timing and location of “sex” determination and differentiation. In some cases, gonadal differentiation may follow determination fairly linearly whereas in others, differentiation may start before determination ends, or both may happen simultaneously (Valenzuela 2008). The spatial and temporal variation in “sex determination” across lineages can be seen in the contrast between mechanisms in which “sex determination” happens first in the “gonads” which then influences the development of “secondary sexual traits” (e.g., Pask and Renfree 2001) to ones in which each cell has independent (autonomous) “sex determination” (e.g., Ioannidis et al. 2021). Furthermore, any initial “trigger” of “sex determination” (e.g., genotypic or environmental) is only one element of a downstream network of genes involved in the sequential, multi-level process of “sex differentiation” (DiNapoli and Capel 2008; Wilhelm et al. 2025). Thus, the steps of determination and differentiation cannot always be neatly separated nor are the traits considered determined during “sexual development” always clear. In addition, rather than being irreversible, “sexual” differentiation requires active maintenance and “sexual” phenotypes, even the “gonad”, can change throughout an individual’s life span (Dranow et al. 2016; Jiménez et al. 2021).

Research on “sex determination” in a comparative evolutionary genetics context places a greater emphasis on the inheritance of factors that influence the development of “sexual” traits in offspring (Valenzuela 2008). This framing has structured the primary division of “sex determination” into two types: those involving inherited loci associated with each “sex type” (genotypic systems) and those where a consistent genotypic association is absent (environmental systems; Valenzuela et al. 2003; Bachtrog et al. 2014). Far from being monolithic, these two categories are extremes of a diverse array of mechanisms that often operate in tandem. Genotypic systems range from polygenic influence to specialized “sex chromosomes,” while environmental systems can be triggered by abiotic factors like temperature or by biotic cues such as local “sex type” ratios and food availability (Bachtrog et al. 2014). In addition, stochastic inputs may also modulate “sex determination” irrespective of the “sex determination” mechanism (Perrin 2016). Lineages with varying levels of plasticity or susceptibility to environmentally induced “sex type” ratio biases demonstrate that “sex determination” systems, as with many traits, contain genetic variation in environmental sensitivity (e.g., Charnov and Bull 1977; Valenzuela 2004; Schwanz and Georges 2021; Krueger et al. 2025). Variation may further be found within and among populations of the same species, which may also reflect evolutionarily transitory states or mixed-strategy systems (Conover and Heins 1987; Perrin 2009; Holleley et al. 2015).

Disciplinary variation in the conceptualisation of designations between “sex determination” and “sex differentiation” reflect how research questions can use the same language with different implications. Conceptual tension can be seen in recent discourses on “polygenic sex determination” with some researchers questioning the prevalence and stability of this type of “sex determination” (e.g., for vertebrates Schartl et al. 2023), and others arguing that the boundaries between polygenic and monogenic chromosomal systems are inherently blurred by the shared logic of their underlying developmental regulatory networks (Kocher et al. 2024). In addition, there can be contextual differences in the meaning of “master sex determining locus” referring in a developmental perspective to a specific active locus at the top of the developmental cascade, whereas in an evolutionary approach the initial trigger can be a general property such as the balance between “sex chromosomes” and autosomes rather than any single dominant locus (Charlesworth 2022). In plants, “sex determination” can have additional contextual variation based on whether differentiation among “sexual” phenotypes occurs at either the “sporophyte” or “gametophyte” life stage (Charlesworth 2002; Jesson and Garnock-Jones 2012; Pannell 2017). Just as with the different ways of conceptualising “sex types,” differences in the subject whose development is being “determined” can result in very different scientific explanations (see also *“Female,” “male” and “hermaphrodite”* section).

#### There are no universal properties of “sex chromosomes” other than a pattern of inheritance associated with “gamete”- producing phenotypes

Enthusiasm for “genotypic sex determination” systems in animals increased with the observation in the early 20th century that visibly dimorphic chromosome pairs in karyotypes were consistent with Mendelian chromosomal models of “sex type” inheritance (Stevens 1905; Wilson 1905). Scientists initially referred to these as “accessory” chromosomes rather than “sex” chromosomes (McClung 1902) and some cautioned against overgeneralization due to the complexity of chromosomal variation in “meiosis” (reviewed in Carey et al. 2022). However, the formulation of “sex chromosomes” coalesced into a simplified binary perspective that extended beyond patterns of inheritance into perception of chromosomes imbued with “feminity” or “masculinity” that contain a “sex determination” gene (Richardson 2013; Charlesworth 2022). A consequence of the intense focus on the association of these chromosomes with “sex” can be an underappreciation of their influence on traits unrelated to “sex” (Xirocostas et al. 2020; Cīrulis et al. 2022). Indeed, in systems with sex determination in the unreduced life stage (i.e., diploid or polyploid; “sporophyte”), one of the “sex chromosome” homologs is found in both “sex types” (Bachtrog et al. 2011). Moreover, the synonymisation of “sex chromosomes” as “genetic sex” can contribute to confusion surrounding the meaning of “sex types" and obfuscate relationships between phenotype and genotype (see also *“Female,” “male,” and “hermaphrodite”* section; Richardson 2013). This tendency can be seen in terminology such as “primary sex ratio” which positions the karyotype as the “real” “sex type” that is altered by the environment during “sex reversal” rather than recognising genotype as only one component of phenotypic developmental pathways. With the advancement of genome sequencing, the relative ease of identifying genotypic differences must be balanced by recognising that they are only one component of “sex determination” and the differentiation and identification of “sex types” also requires investigation via other methods (Stöck et al. 2021; Kocher et al. 2024).

The detection of “sex chromosomes” has further broadened based on evidence from modern genomic studies (e.g., molecular cytogenetics, recombination patterns, genotypic differences, sequence-based signatures of differentiation, association mapping) to include those without obvious morphological or pairing differences designated as “homomorphic” in contrast to “heteromorphic” sex chromosomes (Charlesworth and Mank 2010; Kratochvíl et al. 2021). However, there is no established extent of differentiation that is used to distinguish “heteromorphic” from “homomorphic” “sex chromosome” systems and estimates of differentiation are subject to the sensitivity of the method used (Carey et al. 2022; Charlesworth 2022). Another term sometimes applied to “homomorphic” “sex chromosomes” is “proto-sex chromosome,” which implies an early or “primitive” chromosome at the beginning of an evolutionary process of differentiation or degeneration (Charlesworth et al. 2005). Implications of the designation of “proto” can be misleading, obscuring the range of ages and stability found among “homomorphic” “sex chromosomes” systems. For instance, in some systems “homomorphic” “sex chromosomes” rarely degenerate, such as lineages where “sex chromosome” turnover rates are high (e.g., Gamble et al. 2015; Myosho et al. 2015; Vicoso and Bachtrog 2015; Jeffries et al. 2018), or those in which “sex reversal” contributes to higher rates of “recombination” among “sex chromosomes” (Perrin 2009).

The binary categories of “sex chromosome” types further fails to reflect a process of evolutionary degeneration that “homomorphic”/“proto sex chromosomes” undergo to become “heteromorphic” “sex chromosomes,” or the different processes such as chromosomal fusions (“neo sex chromosomes”) that can contribute to morphological differences (Charlesworth 2022). More critically, the identification of “homomorphic” “sex chromosomes” brings into question the clarity of the term “sex chromosome” itself, which implies that a chromosome is inherited in a “sex type”-associated way in its entirety. Although this may be the case when “recombination” is absent or the “sex chromosome” lacks a homologous pair, instead, for many lineages “recombination” results in a smaller region of the chromosome that is exclusively associated with a “sex type.” Even the highly differentiated “sex chromosomes” of many eutherian mammals contain a small pseudoautosomal region that recombines and is not inherited in strict association with “sex type” (Charlesworth 2021). The region of the chromosome associated with “sex type” may be so small in some lineages, that perhaps they could be more accurately described as “sex determining chromosomes” or chromosomes with “sex-linked regions” rather than “sex chromosomes" (Charlesworth & Mank 2010; Charlesworth 2022).

Independent origins of “sex chromosome” systems, including across taxa which also have independent origins of “anisogamy,” provide exciting opportunities for investigating the factors that influence genome evolution (Charlesworth 2002; Charlesworth and Mank 2010; Bachtrog et al. 2011; Charlesworth 2015; Charlesworth 2019; Mank 2022). “Sex chromosome” systems can differ in numerous attributes (Bachtrog et al. 2011; Furman et al. 2020), including evolutionary trajectory and differentiation, as well as incredible variation in their number (single pair, multiple pairs, or no homologous pair), arrangement (inversions, fusions or fissions), “sex type”-specificity (“sex-limited” chromosome; heterogametic “females” or “males”), and influence on “sex determination” (dominant loci on “sex chromosome,” ratio or dosage of loci on “sex chromosomes” relative to autosomes, and/or interactions with other factors or modifier loci). Nevertheless, remarkable convergence in patterns of genome evolution is observed across different systems. For example, “sex chromosomes” tend to evolve faster and be more involved in species boundary formation than autosomes, regardless of the specific system of karyotype association with “sex type” (Haldane, 1922; Ellegren 2011; Meisel and Connallon 2014; Delph and Demuth 2016). Haploid phase “sex chromosome” systems of algae and bryophytes provide a unique opportunity to compare patterns of “sex chromosome” evolution with plants and animals with diploid stage “sex determination” as well as with “mating type” loci of haploid fungal systems (Coelho et al. 2018; Carey et al. 2021). Similarities in “recombination” suppression between fungal “mating type chromosomes” and plant or animal “sex chromosomes” suggests that the driving processes in “sex chromosome” evolution may correspond to compatibility among individuals rather than differences in “gamete” size (Menkis et al. 2008; Branco et al. 2017; Jay et al. 2024). Similarities in the evolution of “sex chromosomes” and the genomic architecture of polymorphisms (e.g., “supergenes”) or patterns of accumulation of genomic differences in speciation deserve greater attention as theory on “sex chromosome” evolution is developed in response to the changing perception of “sex chromosome” characteristics (Schwander et al. 2014; Charlesworth 2016; Abbott et al. 2017).

### Systems of “a/sexual” individuals

#### Variability in “sex types” is compounded in the description of “sexual systems”

Descriptions of “sexual systems” are often focused on naming the individuals whose “gametes” contribute to the next generation. For example, “sexual system” types often don’t reflect when reproductive success involves multiple individuals, whether for offspring survival or for genetically compatible “sexual” reproduction in the next generation. In addition, the focus on “sexual reproduction” restricts the recognition of individuals, which may contribute to the next generation through “asexual reproduction” (see also *“A/sexual reproduction”* section). Nevertheless, even just describing systems based on the configurations of “gametic” contributions can be difficult because the number of different possible configurations of individuals can increase rapidly as a result of factorial combinations of the different “sex types” that are present in a given lineage (see also “Female,” “male,” and “hermaphrodite” section). Different terminology for “sexual systems” in which individuals produce only one type of “gamete” is an example of how language can also communicate other interacting traits of lifestage and modularity. Specifically, “gonochorism” is predominantly used for unitary (unreduced) animals, “dioecy” for modular (unreduced, “sporophyte”) plants, and “dioicy” for unitary (haploid or reduced, “gametophyte”) plants (Wyatt 1985). Descriptions of “sexual systems” in land plants further incorporate a combination of the expression within a modular reproductive structure as well as across different reproductive structures. The challenges of naming “sexual systems” reflects the compounding complexity of describing “sex types” at the individual level.

Variation in the morphology or “sex types” among individuals found in a lineage can further complicate simple categorisation of “sexual systems” (see *“Female,” “male,” and “hermaphrodite”* section). For instance, bryophyte systems of classification have suggested additional terminology for “dioicious” systems based on whether the “male” is reduced in form and living either independently (“heterodioicious”) or epiphytically on the stem (“cladodioicious”) or leaves (“phyllodioicious”) of the “female” plant (Wyatt 1985). The close association of individuals may mistakenly appear “hermaphroditic”. A similar misclassification can occur in angiosperm plants with “cryptic dioecy” in which individuals may appear to be “hermaphroditic,” with both “pollen” and “ovule” structures, but “reproduction” can only occur through one type of “gametophyte” (Mayer and Charlesworth 1991). Conversely, it is also not uncommon to find infrequent occurrences of “hermaphroditic” individuals in angiosperm plants with “dioecious” “sexual systems.” This “leaky dioecy” or “subdioecy” is hypothesized to be a mechanism of “reproduction” assurance if plants grow in an area with no nearby compatible individuals and can lead to a transition to “hermaphroditic” “sexual systems” (Ehlers and Bataillon 2007; Cossard et al. 2021).

Mixtures of different “sex types” that do not neatly conform to pre-defined categories of "sexual systems" have been incredibly useful in investigations of “sexual system” evolution (Pannell and Jordan 2022). Notably, there are many different transitions in “sexual systems” possible between “hermaphroditism” and “dioecy” or “gonochorism” (Barrett 2002; Pla et al. 2022; Lesaffre et al. 2024). While “gynodioecy” (system of “hermaphrodite” and “female” individuals) is a frequent “intermediate” “sexual system” found in plants and “androdioecy” (system of “hermaphrodite” and “male” individuals) is a less common “intermediate,” the opposite pattern is found in animals (Pannell and Jordan 2022). The evolutionary trajectory that results in “sexual systems” of “dioecy” or “gonochorism” can contribute to different traits of “female” and “male” “sex types” across lineages (Barrett 2002; Lesaffre et al. 2024). Correspondingly, the mechanisms of “sex determination” and “sex types” present in a population can in turn influence the evolution of “sexual systems” in a lineage (Leonard 2018). Notably, transitions among “sexual systems” appear to be reversible, reflecting not only adaptive responses but also phylogenetic and genetic constraints, as well as other facilitating factors, such as gonadal plasticity (Pla et al. 2022). These transitions are associated with selection pressures related to “inbreeding,” “reproductive” assurance, population structure that influence “mating” opportunities, mechanisms of “sex determination,” and developmental plasticity of “reproductive” structures (e.g., in fish Benvenuto et al. 2017; Pla et al. 2022 and invertebrates Weeks 2012, reviewed in Pannell and Jordan 2022). Thus, although “sexual systems” may specifically describe patterns of “gamete” production across individuals, the evolutionary transitions among “sexual systems” also involves selection related to intrinsic and extrinsic factors that influence “reproductive” success (Schwander et al. 2014; Leonard 2018; Scott et al. 2018).

#### Many traits influence the compatibility for individuals to “sexually reproduce” with themselves or others, influencing the genetic diversity of offspring

Incorporating all the different factors that influence which individual(s) reproduce together in “sexual systems” involves accounting for a wide variety of traits beyond “gamete” production (see also *“Female,” “male,” and “hermaphrodite”* section) inconsistently labeled within and between taxa as “mating systems” or “breeding systems” (e.g., in plants Neal and Anderson 2005). Incompatibilities can be phenotypic, corresponding to spatial, morphological, behavioral, or temporal barriers for contact between “gamete” types, or genetic interactions that influence compatibility during “gamete” transport or fusion (Clo et al. 2025). In describing the framework of terminology related to the “reproductive system” of angiosperms, Cardoso et al. (2018) included not only “sexual systems,” but also “sporophytic” and “gametophytic” traits of “floral systems,” “incompatibility systems,” and “mating systems.” Each of these systems includes a variety of possible states, for instance “self incompatibility” can range from differentially permissive or “leaky” to instances of “self-fertilisation” creating continuous variation rather than binary categories of “outcrossing” and “selfing” (Vogler and Kalisz 2001; Goodwillie et al. 2005; Whitehead et al. 2018). Notably, not all plant systems have received equal attention and obligate “outcrossing” (including “dioecy”) in angiosperms in particular is undersampled (Igic and Kohn 2006; Meyer et al. 2025).

As in plants, “hermaphroditic” animals have been found to vary in the relative rates of “selfing” and “outcrossing” (Jarne and Charlesworth 1993; Jarne and Auld 2006; Escobar et al. 2011). In addition, in “gonochoristic” animals traits such as the number of “mates,” “sexual behaviors,” “mating resources,” “mate choice,” “pair bonds,” and “parental” care can influence “reproductive” outcomes (Emlen and Oring 1977; Reynolds 1996). Without “anisogamy,” fungal “sexual systems” primarily describe the types of “sexual reproduction” possible, either only “outcrossing” between different “mating types” (“heterothallic”), or both “outcrossing” and “selfing” with the same or different “mating type” (“homothallic”) (Esser 1971; Sun et al. 2025). However, fungal “mating” compatibility can also be influenced by additional properties such as “mating type” switching, number and linkage of “mating type” loci (e.g., bipolar or tetrapolar), and the number of haploid nuclei in a cell (Billiard et al. 2012; Taylor et al. 2015; Yadav et al. 2023). Across species, “sexual” interactions in populations may be better represented as quantitative measures of “reproductive” success among individuals rather than systems based on categories of “sex type” or “mating type” (Kokko et al. 1999; Bertram and Gorelick 2009; Benvenuto and Lorenzi 2023).

**Fig. 1:**
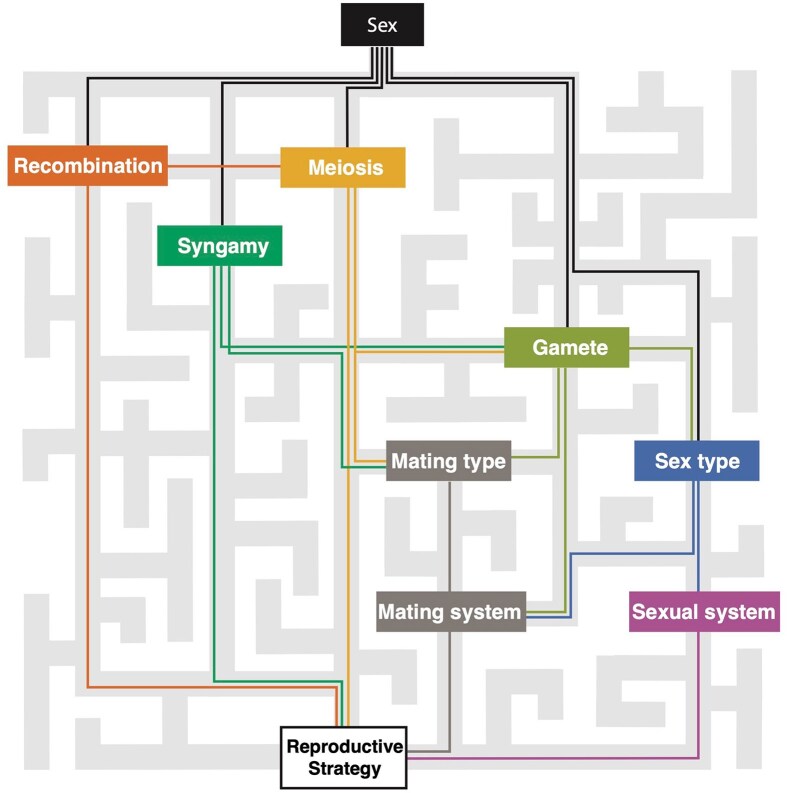
We construct a labyrinth as a way to navigate the interacting traits of “sex” that contribute to a "reproductive strategy". The evolution of these traits influence each other as well as the possible reproductive outcomes. For example, genetic diversity of offspring can vary among reproductive strategies, influenced by the specific characters of traits in a system. Navigating the labyrinth involves considering how traits directly, and indirectly, associated with “sex” including cellular processes (“recombination,” “meiosis,” or “syngamy”), cell types (“gamete”), traits of individuals (“mating type” or “sex type”) and the interactions within and among individuals (“mating system” or “sexual system") contribute to properties of a "reproductive strategy". Mapping “sex” as a compound concept facilitates making comparisons across lineages based on the specific traits expressed and how they interact with each other.

A focus on the impact of traits on “reproductive” outcomes, rather than the trait itself, can facilitate cross-taxa comparisons (Fig 1; see also *Navigating meanings of “sex”* section). In particular, an analysis from Clo and collaborators (2025) united “mating systems” concepts in plants (i.e., rate of “self-fertilization”) and animals (i.e., formation of “mating pairs”) with respect to their influence on the genetic variation in the next generation. By connecting terms developed in plant and animal contexts through their relationship to “inbreeding” and spatial dynamics, Clo et al. (2025) demonstrate how cross-taxa comparisons can refine and expand research on “mating system” evolution. Formulating “mating systems” with respect to compatibility further allows for comparisons to be made on the impacts of different forms of “self-fertilization” on “inbreeding” in species without “anisogamy” (Billiard et al. 2012; Krueger-Hadfield 2024; Nieuwenhuis and McDaniel 2025). For example, the possible crosses in “heterothallic” “isogamous” organisms can resemble “outcrossing” (i.e., “gonochorism” and “dioecy”) or diploid “intergametophytic selfing” (i.e., “dioicy,” “monoecy,” “hermaphroditism”) in “anisogamous” organisms, whereas, the possible crosses in “homothallic” “isogamous” organisms also include haploid “intragametophytic selfing” (“monoicy”) (Krueger-Hadfield 2024). Moreover, the potential for both intra- and inter-tetrad haploid “sexual reproduction” presents opportunities for nuanced comparisons with “parthenogenetic” alterations in “meiosis” on genetic variation of the next generation (see also *“A/sexual reproduction”* section; Billiard et al. 2012; Jaron et al. 2021). Finding connections across taxa-specific terminology can ultimately provide greater insight into how factors such as ploidy and inbreeding influence evolution of “reproductive” modes across individuals (Cutter 2019; Olsen et al. 2021; Clo et al. 2025).

#### Conception of “sexual systems” depends on how the group of individuals is perceived and can include multispecies interactions

Finally, categorising systems of individuals involves a delineation of which individuals are a part of a lineage. Recognizing the size and the structure of a “group” (often a population or "species") of individuals is a critical component of understanding the systems found among individuals in their environment (West-Eberhard 1983). As the variation considered to be part of a “sexual system” is bounded by when individuals are considered sufficiently diverged to constitute a new “species,” the recognition of “sex types” and “species” are entangled. For instance, the mixture of phenotypes and alterations to “reproductive” functions in interspecific hybrids can set them outside the boundaries of “sexual systems.” Furthermore, “sexual systems” that involve multispecies interactions, as is frequently found in animal “pollination” in plants or in endosymbiont effects on insect “reproduction,” do not fit into typical concepts of “sexual systems” pertaining to a singular lineage and require a more expansive understanding of the components of these systems (Barrett and Harder 2017; Fricke et al. 2025). The many theories for the evolution and maintenance of “sexual reproduction” (or lack thereof) that involve interactions with parasites or environmental conditions further indicate that “sex” occurs within a “multispecies” ecological framework (Otto and Nuismer 2004). Conceiving of all the components of an “a/sexual system” broadens and complicates what traits are considered relevant to “reproduction.” Engaging in this process can improve cross-taxa comparisons and lead to more comprehensive understandings of “sexual” evolution.

## Navigating meanings of “sex”

The meaning of “sex” in any instance is impacted by the assumptions made about the properties of traits within an interconnected network. Approaching “sex” as a labyrinth serves as a useful framework for revealing the relationship among traits that contribute to the totality of a reproductive strategy within a lineage (Fig 1). For example, connections of “sex type” with “gamete,” “sexual system” and “mating system” represent how the “reproductive” opportunities of an individual are influenced not only by properties of “gametic” compatibility but also phenotypic or genetic compatibility within one or among multiple individuals (see also *“Female,” “male” and “hermaphrodite”* and *Systems of “a/sexual” individuals* sections). In addition, the use of “sex types” for both “sexual” and derived “asexual” or “parthenogenetic” lineages is clarified by their shared connection to “gamete” even though the process of “meiosis” and “syngamy” may be altered (see also *“A/sexual reproduction”* section). Furthermore, connections reveal the potential for traits to influence each other, such as in the relationships between mechanisms of “sex determination” and the evolution of “sexual systems” (see also *Systems of “a/sexual” individuals* section). Thus, the structure of a labyrinth can support the dissection of how concepts are variably constructed across taxa and disciplines (Fig 1; Table 1).

The structure of a labyrinth can be used to clarify investigations into the contribution of the component traits of “sex” to a specific research question. For example, although the evolution and maintenance of “sex” is often associated with generation of genetic diversity, this property can be highly variable among “sexual” and “asexual” lineages (see also *“A/sexual reproduction”* section). The process of disambiguation through the labyrinth framework reveals the many traits of “sex,” such as mating systems (including self-compatability and opportunity for outbreeding), presence or timing of meiosis, and extent of recombination, which may each modify and influence the potential genetic diversity of offspring (see also *Systems of “A/sexual” individuals”* section; Billiard et al. 2012; Krueger-Hadfield 2024; Clo et al. 2025). Aspects of reproductive strategies related to phenotypic variation could similarly be investigated with greater nuance by identifying multiple interacting component factors rather than fixating on one trait such as gamete size (Gourevitch et al. 2026). Being aware of which traits are implicated in any specific usage of “sex” can facilitate identifying the influence of a specific construction of “sex” on the formulation of research questions and interpretations of data (Richardson 2022, Ritz and DuBois 2025).

An example of the consequence of alternate meanings of “sex” in the formation of scientific research questions can be seen in disagreements over how to define “sexual selection” (e.g., West-Eberhard 1983; Alonzo and Servedio 2019; Shuker and Kvarnemo 2021; Janicke 2024). Arguments for definitions that restrict “sexual selection” specifically to differential access to “gametes” adhere to an “anisogamy” definition of “sex types.” In contrast, arguments that “sexual selection” should also include other factors that influence “reproductive” outcomes are consistent with more expansive definitions of “sex types” as dynamic systems that include many different traits. Recognising that different concepts of “sex types” are being referenced in conflicting definitions of “sexual selection” demonstrates how the meaning of “sex” can have radiating consequences for the structure of scientific thinking. The labyrinth framework also facilitates considering how other components of the meanings of “sex,” such as compatibility and interactions among individuals, may contribute to processes such as “sexual selection.” For example, models of “sexual selection” can be described by “reproductive” dimorphism rather than requiring a specific reference to “gametes” or “sex types” (Evron 2024). In addition, incorporating ecological factors that influence “mating systems” is critical for a more comprehensive understanding and appreciation of the evolution of phenotypic variation related to “reproductive” and “sexual” traits (Gourevitch et al. 2026). Engaging with “sex” as a labyrinth of concepts may also be useful when considering the application of “sexual selection” theories developed in unitary, unreduced, “gonochoristic” animal systems to plant, fungi, or hermaphroditic animal systems. Identifying the assumptions about “sexual reproductive” cycles or compatibility due to “sexual systems” and/or “mating systems” embedded within theories is a crucial step in evaluating how these properties may differ among lineages (e.g., Leonard 2006; Beekman et al. 2016; Tonnabel et al. 2026).

The immense remaining challenge is how to accurately communicate the specific intended meaning within the labyrinth of “sex.” A starting place can be increased precision in the specific traits used to define categories of “sex types” in research studies (Richardson 2022; Massa et al. 2023; McLaughlin et al. 2023; Smiley et al. 2024; Thinius and Trappes 2024; Eppely et al. 2026). Notably, examples of more precise language and descriptions of concepts that have been developed in the context of more inclusive science teaching can also apply to research (Hales 2020; Long et al. 2021; Zemenick et al. 2022; Casper et al. 2025). Comparisons across lineages can also be informative for identifying the similarities and differences in the polysemous language used to describe "sexual" and "reproductive" traits (Meissner 2021; Sandford 2022; Subramaniam and Bartlett 2023; Warkentin et al. accepted, Young et al. in preparation). Critically, the classification of “reproductive” characters and their interrelationships can influence how these traits are investigated in scientific research (Córtes-García et al. 2024).

Systematic comparisons across taxa requires establishing agreed upon meaning of traits and their relation to each other (Bachtrog et al. 2014; Jeffries et al. 2025). Structured ontologies from information sciences are a tool to codify the knowledge in any specific field and may be useful in clarifying and formalising the relationships among concepts of “sex” (Jeffries et al. 2025). For example, an ontology of standardized language for “reproductive” isolation has been developed as a tool to facilitate comparisons across the variable terminology of “speciation” research (Stankowski et al. 2024; Walker et al. 2026). Entries in ontologies represent fixed, singular concepts whose meaning is specified by their relationships to other concepts which can include restrictions to specific conditions such as applicability to certain taxa (Smith et al. 2007; Jackson et al. 2021). The Tree of Sex 2.0 consortium is developing an ontology to structure a database on “reproductive” traits that can facilitate comparisons across eukaryotes (Jeffries et al. 2025). The ongoing process of building the ontology (https://github.com/Tree-of-Sex/ToS-Ontology) can be observed as part of the scientific record and is a dynamic format that can change in response to input. The formation of a “sex” ontology can be a resource to navigate the contextual language used to conceptualise and communicate organismal traits.

## Conclusion

In questioning and struggling with the many different meanings of “sex,” we have established how the context in which the language of “sex” emerges has influenced how it is understood and used in scientific research. For example, there is a shared interest across research in “a/sexual reproduction,” “sexual systems,” “mating systems” and “sex types” on the impact “sex” can have on genetic variation. Similarly, research on mechanisms of determination or differentiation can be applied to the many different attributes of “sex” both as phenotypic polymorphisms as well as modes of “reproduction.” Moreover, the investigation of evolutionary transitions among the types of individuals and modes of “reproduction” in systems requires integration of all of the different component traits of “sex.” In constructing a labyrinth of “sex,” we are better able to see how seemingly distinct concepts are instead interconnected parts that influence and co-create each other to form various concepts of “sex.” Engaging with the full complexity of “sex” can be a tool for destabilising prevailing associations of “sex” with binary types of “gametes” in “anisogamy.” The multiplicity of “sex” can be approached not as an error or an argument but as an opportunity to learn more about the extraordinary variation in the natural world, the scientific process, and the cultural contexts that shape scientific knowledge.

## Notes

Tree of Sex Consortium (in alphabetical order): Astrid Böhne, Veronika Bókony, Thomas Desvignes, Duminda S. B. Dissanayake, Daniela Palmer Droguett, Tony Gamble, Louise D. Heitzmann, Mark S. Hibbins, Fredric J. Janzen, Kamil S. Jaron, Ravinder K. Kanda, Caleb J. Krueger, Joris M. Koene, Lukáš Kratochvíl, Máire Ní Leathlobhair, Wen-Juan Ma, Cristiana Ramalho Maciel, Stuart F. McDaniel, Zoran Marčić, Ashwini V. Mohan, John R. Pannell, Brendan J. Pinto, Giacomo Potente, Wagner Luiz dos Santos, Mércia Patrícia Pereira Silva, Péter Szövényi, Ricardo Utsunomia, Jie Wang, Melissa A. Wilson, Charlotte J. Wright.

From the symposium “Sex Across Origins: Questioning Animal-centric Assumptions and Developing Integrative Frameworks” presented at the annual meeting of the Society for Integrative and Comparative Biology, January 3-7th, 2026. 
